# Validation of the child oral health impact profile (COHIP) french questionnaire among 12 years-old children in New Caledonia

**DOI:** 10.1186/s12955-015-0371-9

**Published:** 2015-10-30

**Authors:** Nada El Osta, Helene Pichot, Delphine Soulier-Peigue, Martine Hennequin, Stephanie Tubert-Jeannin

**Affiliations:** Clermont University, University of Auvergne, EA 4847, Centre de Recherche en Odontologie Clinique, BP10448, F-63000 Clermont-Ferrand, France; Department of Public Health, Saint-Joseph University, Beirut, Lebanon; Sanitary and Social Agency of New Caledonia, Nouméa, New Caledonia; CHU Clermont-Ferrand, F-63003 Clermont-Ferrand, France

**Keywords:** Oral Health related Quality Of Life, Children, New Caledonia, Ethnicity

## Abstract

**Background:**

The Child Oral Health Impact Profile (COHIP) is an Oral Health Related Quality of Life (OHRQOL) tool that assesses the impact of oral diseases on quality of life in children. This study aimed to assess the validity of the COHIP French questionnaire (45 items) and to evaluate the OHRQOL of 12-years children in New Caledonia.

**Methods:**

After cultural adaptation of the COHIP questionnaire, data were collected from clinical oral examinations and self-administered questionnaires in a representative sample of children aged 12 years in New Caledonia. Questions related to socio-demographic status or children’s perception of their oral and general health were added to the COHIP questionnaire. Studying the association between COHIP scores and health subjective perceptions or dental status indicators assessed concurrent and discriminant validity. The items of the COHIP were subjected to principal components analysis. Finally, reproducibility and reliability were evaluated using Intraclass Correlation Coefficients (ICC) and Cronbach’s alpha coefficient.

**Results:**

Two hundred and thirty-six children participated in the main study; mean age was 12.6 ± 0.31 years, 55.1 % were girls and diverse ethnic groups were represented. A preliminary reliability analysis has led to calculate COHIP scores with 34 items as in the English version, scores ranged from 35 to 131 (mean ± SD, 101.9 ± 16.84). Lower COHIP scores were significantly associated with the self-perception of poor general or oral health. COHIP was able to discriminate between participants according to gender, ethnic group, oral hygiene, dental attendance, dental fear and the presence of oral diseases. Test–retest reliability and scale reliability were excellent (ICC = 0.904; Cronbach’s alpha coefficient = 0.880). Four components were identified from the factor analysis.

**Conclusion:**

The French 34-items COHIP showed excellent psychometric properties. Further testing will examine the structure and utility of the instrument in both clinical and epidemiological samples.

## Background

It has been recently reported that one in two 12-year-old children in New Caledonia (NC) was affected by dental caries with at least one untreated cavitated lesion [1]. As in other developed countries of the Pacific, socio-economic status and ethnicity had a strong influence on dental status leading to high oral health disparities [1–3]. Thus, implementing effective oral health promotion interventions is necessary in this overseas French territory, especially for children and adolescents. To this end, accurate oral health indicators are needed to help the definition of the objectives of those interventions and to assess their future impact.

In order to evaluate oral health, the measurement of oral health-related quality of life (OHRQoL) is needed in addition to the use of dental status indicators [4, 5]. Indeed, subjective OHRQoL measures and normative dental indicators, even if associated, are of different nature. They must be combined in order to cover the different dimensions of oral health for public health decisions.

The impact of dental status on children’s health and well-being has been widely demonstrated [6–8]. Dental diseases can lead to functional but also psychological disorders affecting children’s quality of life [6]. In particular, the pain and sometimes lack of sleep generated by the presence of untreated dental caries may have negative impacts on children’s attention at school [7]. Measuring the impact of dental status on well being also helps in justifying the cost of oral health promotion (OHP) programs. In this area, OHRQoL questionnaires are useful tools, as they are able to detect positive changes in oral health after dental treatment or OHP programs [8–11].

In the past fifteen years, several specific OHRQoL instruments have been developed for evaluation in children and adolescents: CPQ [12, 13], CPQ 11–14 [14], child-OIDP [15], ECOHIS [16], COHIP [17–19]. Some of them, such as the Child-OIDP have already been validated in France [20].

Among all, the Child Oral Health Impact Profile (COHIP) was developed using a multi-staged impact approach with an initial pool of 54 items chosen after a literature and expert review [12]. Experienced translators translated all the items into French and Spanish. Linguistic equivalence across the various language versions was achieved continuously during the validation process. Then face validity and item impact were evaluated in four sites including a French-speaking one (Montreal, Canada). Thus, the final English questionnaire consisted of 34 items and five subscales: oral health, functional well being, social/emotional well-being, school environment and self-image [17, 18].

The COHIP has shown good psychometric properties in different community samples [19, 21–23]. It has already been tested in various clinical conditions among children with craniofacial conditions or orthodontic needs and in different countries including US, Korea, Iran and the Netherlands [22, 24–26]. The COHIP incorporates a specific dimension (the School Environment dimension) particularly well suited for the evaluation of OHRQOL in 8–15 year old children. It has also been shown that the COHIP is able to discriminate between groups depending on their experience of dental decay and their perceptions about appearance. Thus, this instrument seems relevant for child populations with a high prevalence of dental problems. Finally, the COHIP includes questions that measure the positive aspects of oral health [4, 27].

Since its initial development by Broder et al., the COHIP has been translated in various languages for use in different places and cultures. However, the French version of the COHIP questionnaire (45 questions) has been preliminary tested in a sample of 35 children aged 9–14 years in 2002 but has never been used nor tested in a community sample [28].

The main goal of this study was thus to test the reliability, reproducibility, convergent and discriminant validity of the COHIP French questionnaire in a community sample. This study also was intended to explore the structure of the French COHIP questionnaire within the cultural context of New Caledonia. In addition, the aim was to evaluate among 12-year-old children the OHRQoL in New Caledonia before implementation of an OHP program.

## Methods

### Samples and validation process

**Fig. 1:**
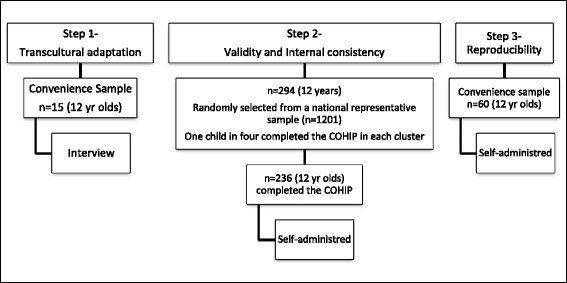
Description of the samples

First, clarity of the wording of the COHIP items was assessed in a convenience group of 15 New Caledonian children of different ethnic origins (Fig. 1). Children indicated whether each of the items was clear or unclear and if the format was readable enough for them. A slight adaptation of the content of the French COHIP questionnaire was done accordingly.

Second, the validity of the COHIP questionnaire (concurrent, discriminant validity, internal consistency) was evaluated in a representative sample of 12-year-old New Caledonian children. A national dental epidemiological survey had been conducted in 2012 in New Caledonia among a representative sample of 1201 children aged 12 years [1]. The children had been clinically examined within this national study and one child in four within each cluster (=school) was asked to complete the COHIP questionnaire (294 children).

Finally, reproducibility was assessed by repeating the administration of the COHIP using a convenience sample of 60 12-year-old schoolchildren in the city of Noumea. Children were recruited in three schools that participated in the national survey and where the distributions of the COHIP scores were similar to that of the national sample. Test–retest participants were included only if the participants said no change in general or oral health status had occurred since their first test. The test-retest procedure was conducted at a later period, thus children from the main representative sample mentioned above could not be interviewed.

### Compliance with ethical standard

In the absence of a single New Caledonian ethical committee when the study was conducted, ethical approvals were obtained from the New Caledonian educational and health institutions. Schools were approached through local educational authorities. Explanatory letters and consent forms were sent to parents a few days prior to the dental examination or interview and only those children whose parents returned written consent were included. Written parental consent and each child’s verbal consent were obtained for all the participants.

### COHIP questionnaire

The COHIP French questionnaire consists of 45 items representing several theoretical domains [22]: (i). “Oral health”: ten negatively worded questions evaluate specific oral symptoms (e.g., pain, spots on teeth). (ii). “Functional well-being”: eight questions relate to the child’s ability to carry out specific everyday tasks or activities (e.g., speaking clearly, chewing). (iii). “Social-emotional well-being”: 13 negatively worded items concerning peer interactions and mood states. (iv) “School environment” with four negatively worded items evaluating tasks associated with the school environment. (v) “Self-image”: eight questions addressing experience (e.g., been confident, felt attractive) and feelings. (vi) “treatment expectations” : two questions.

### Scoring of the COHIP questionnaire

Children rated whether they had “almost all of the time”, “fairly often”, “sometimes”, “almost never” or “never” experienced in the past three months any of the situations listed. Responses were scored on a scale ranging from 0 (almost all the time) to 4 (never) with a higher score indicating satisfactory OHRQoL. Positively worded scores were reversed for the calculation of the global score. Additionally, for the positively worded items regarding self-feelings, the response set includes 0 = ‘strongly disagree’; 1 = ‘somewhat disagree’; 2 = ‘don’t agree or disagree’; 3 = ‘somewhat agree’; and 4 = ‘strongly agree’ with a lower score indicating poorer oral health perception.

Subscale scores were computed as the sum of the responses on that subscale. The overall COHIP score was computed by summing the subscale scores and ranged from 0 to 180. If more than two-thirds of the items in a subscale were missing, the scores were not computed. If fewer than two-thirds of the items in a subscale were missing, the average of available items was used to replace the missing items and the total scores were calculated.

### Complementary variables

Within the national epidemiological survey, many data items were collected during clinical oral examinations and through the administration of questionnaires to the children. This information was used in the present validation study with a few supplementary questions related to general health and satisfaction with oral health.

#### Questionnaires

The questionnaires included socio-demographic variables (gender, date of birth, ethnic group and school attendance) and questions relating to satisfaction with oral health, perception of own general health, dental problems and need for dental care. Children also answered questions relating to dental attendance (had never or already had visited a dentist) and dental fear level (Visual Analogic Scale (VAS) from 0 = no fear, to 10 = severe fear) [29].

#### Clinical examinations

The presence of a dental infectious process (presence of an abscess, a tooth with pulpal exposure, a fistula), a mucosal lesion on lips, tongue or jaw (traumatic lesions, ulcers or bites) and untreated fractured permanent anterior teeth were recorded. Radiographs were not used and carious lesions were diagnosed at the dentinal threshold (D3) on permanent teeth [30, 31]. The number of decayed, missing or filled permanent teeth (D_3_MFT) was calculated [31]. The Greene & Vermillion oral hygiene index [32] was used to record the presence of calculus and dental plaque and the scores were dichotomised: absence (score 0 for all sextants) or presence of calculus or dental plaque (score >0 for at least one sextant). The Gingival Index of Löe & Silness [33] was used to express the gingival status, and the scores were trichotomised: no gingivitis (score 0 for all sextants), has localised gingivitis (score >0 for at least one sextant of one arch) or extensive gingivitis (score >0 for all sextants of at least one arch). Additionally, the children’s level of need for dental treatment was evaluated with the Clinical Oral Care Needs Index (COCNI), developed in a previous survey [34]. In this index, clinical oral indicators and subjective variables are associated within an algorithm using logical association (If…then… else…) to produce 4 levels of treatment need. Level 1, the child has risk factors for oral diseases (presence of dental plaque, absence of dental attendance…). Level 2, the child has signs of oral diseases (presence of localised gingivitis or at least one untreated fractured permanent anterior tooth or at least one carious lesion involving dentine…) and requires treatment. Level 3, the child requires urgent treatment because signs of focal infectious disease are present (presence of at last one dental infectious process or mucosal lesion or presence of extended gingivitis or acute dental pain). Level 0, the child has no need for either oral examination or treatment.

#### Data collection

For the transcultural adaptation, face-to-face interviews were conducted with the children. The COHIP questionnaires were self administered at school for the main validation process and for the test-retest procedure. Six calibrated dental practitioners performed the children’s examinations within the main validation sample at school [1]. The children completed the complementary questionnaires by themselves the day of the dental examination.

### Statistical analyses

Internal consistency was evaluated by calculating Cronbach’s alpha and alpha, if an item was deleted. The degree of internal consistency was evaluated for the COHIP global score as well as for each subscale. The acceptable level for the overall scale was set at 0.80.

Since there is no gold standard for OHRQoL indices, the validation process relies on the evaluation of construct validity.

Concurrent validity examines a logical hypothesis by testing the index against a proxy measure of a similar concept. It was hypothesised that subjects with lower COHIP scores would be less satisfied with their oral health, would have poorer self-rated oral and general health and would report more treatment needs. As the scores derived from the COHIP were not normally distributed, Mann–Whitney and Kruskal-Wallis tests were used.

The relationship between socio-demographic or behavioural variables, dental status and the COHIP scores were also studied. It was hypothesised that patients with a poorer dental status (presence of decayed teeth, calculus, dental plaque, gingivitis, infectious dental processes, mucosal lesions, untreated fractured permanent anterior teeth and children with treatment needs…) would have lower COHIP scores. The COHIP score was also supposed to vary between participants with different socio-demographic characteristics (sex, ethnic group, school attendance) and dental behaviours (dental attendance, dental fear). Discriminant validity was explored by measuring the degree to which COHIP scores and clinical data were related using the Spearman correlation coefficient.

The items of the COHIP were subjected to principal components analysis (PCA). Prior to performing PCA, the suitability of data for factor analysis was assessed. Inspection of the correlation matrix revealed the presence of many coefficients of 0.3 and above. The Kaiser-Meyer-Oklin value was 0.858, exceeding the recommended value of 0.6 [35] and the Bartlett’s Test of Sphericity reached statistical significance, supporting the factorability of the correlation matrix [36]. The number of components was assessed using the principal component analysis with eigenvalues exceeding 1, the inspection of the screeplot results and the parallel analysis with eigenvalues exceeding the corresponding criterion values for a randomly generated data matrix of the same size (34 variables and 236 respondents) [37].

Reproducibility was measured using the data on the 60 children who were re-interviewed 1 week after the first evaluation. The Intraclass correlation coefficients (ICC) for the global scores and for each domain were calculated with a two-way random effects model. Acceptable test–retest reliability rating was set at 0.70.

## Results

In all, 311 children participated in the study: 15 children for the transcultural adaptation phase, 236 for the main validation process and 60 for the test-retest evaluation. Indeed, 236 children completed the questionnaire adequately and were thus included in the main validation study (age ± SD: 12.02 ± 0.31 years). The 58 non-participating children were mainly children who partly completed the COHIP questionnaire with more than two-thirds of missing items per subscale.

### Transcultural adaptation (*n* = 15)

No major change was made in the questionnaire’s format, the content of the items or the wording, but the French translation of the self-image’s domain response set (‘strongly disagree’; ‘somewhat disagree’; ‘don’t agree or disagree’; ‘somewhat agree’; and ‘strongly agree’) was modified to improve children’s comprehension.

### Item reduction (*n* = 236)

The full version of the French COHIP questionnaire (45 questions) was used in NC main sample but preliminary analyses (item-score correlation, alpha Cronbach if item deleted, factor analysis) showed that it was preferable to calculate the scores based on the 34-items English version. In the Functional well-being dimension, two positively worded questions (able to keep teeth clean and able to eat all kind of foods) were deleted while the similar worded negatively questions were preferred. In the self-image dimension, a negatively worded question was deleted (Felt that you are not looking good) and the positively worded question preferred. The items-score correlations were low and Cronbach-alpha improved when those items were deleted. Similarly, in the Psychological and social well-being and self image dimensions, respectively five items (Being left out, Asked questions by others, Upset with being asking questions, Avoided meeting new people, Argued with other children) and one item (I am happy with my smile) showed low items- score correlations and were removed. In the treatment expectations dimension, two items (feel better when treatment is completed, nervous about the treatment that I need) were deleted due to the low response rate (respectively 18 and 20 % of non response).

The overall COHIP score was thus computed for the 34 remaining items by summing the subscale scores (Oral Health: 0–40; Functional well-being: 0–24; Social-emotional well-being: 0–32; School environment: 0–16; Self-image: 0–24) and ranged from 0 to 136.

### OHRQOL of the New Caledonian children (*n* = 236)

The proportion of girls was 55.1 %. Self-reported ethnicity revealed that 23.6 % of the children identified themselves as Kanak/Melanesian, 7.9 % as European or Asian, 51.3 % as Caledonian and 17.2 % as Polynesian. The main socio-demographic characteristics did not differ when compared with the sample of 1201 children from the national epidemiological study (1). The COHIP scores ranged from 35 to 131, mean ± SD = 101.9 ± 16.84. According to COHIP, 96.2 % of the children had experienced oral health problems in the previous three months, 72.9 % had functional problems, 60.2 % had socio-emotional impacts, 31.8 % had school or environmental impacts and 92.2 % had self-image impacts (Table 1). Oral diseases were frequent in the study population: 47 % of the children had at least one untreated carious lesion and 25 % had recently experienced dental pain.

**Table 1:** Frequency distribution (n (%)) of the responses for COHIP items (*n* = 236)

In the past three months, have you…	Almost all the time	Fairly often	Sometimes	Almost never	Never
Domain 1: Oral health					
Q1: Had pain in your teeth/toothache	4 (1.7 %)	10 (4.2 %)	78 (33.1 %)	36 (15.3 %)	108 (45.8 %)
Q2: Been breathing through your mouth or snoring	12 (5.1 %)	22 (9.3 %)	59 (25.0 %)	32 (13.6 %)	111 (47.0 %)
Q3: Had discoloured teeth or spots on your teeth	11 (4.7 %)	14 (5.9 %)	50 (21.2 %)	34 (14.4 %)	127 (53.8 %)
Q4: Had crooked teeth or spaces between your teeth	19 (8.1 %)	24 (10.2 %)	50 (21.2 %)	34 (14.4 %)	109 (46.2 %)
Q5: Had sores/sore spots in or around your mouth	3 (1.3 %)	15 (6.4 %)	38 (16.1 %)	25 (10.6 %)	155 (65.7 %)
Q6: Had bad breath	13 (5.5 %)	18 (7.6 %)	85 (36.0 %)	61 (25.8 %)	59 (25.0 %)
Q7: Had bleeding gums	12 (5.1 %)	26 (11.0 %)	63 (26.7 %)	27 (11.4 %)	108 (45.8 %)
Q8: Had food sticking in or between your teeth	27 (11.4 %)	46 (19.5 %)	99 (41.9 %)	38 (16.1 %)	26 (11.0 %)
Q9: Had pain or sensitivity in teeth with hot/cold things	23 (9.7 %)	31 (13.1 %)	63 (26.7 %)	28 (11.9 %)	91 (38.6 %)
Q10: Had dry mouth or lips	22 (9.3 %)	48 (20.3 %)	99 (41.9 %)	30 (12.7 %)	37 (15.7 %)
Domain 2: Functional Well-Being					
Q11: Had trouble biting/chewing apple, carrot/firm meat	7 (3.0 %)	12 (5.1 %)	43 (18.2 %)	33 (14.0 %)	141 (59.7 %)
Q15: Had difficulty eating foods you would like to eat	4 (1.7 %)	5 (2.1 %)	39 (16.5 %)	22 (9.3 %)	166 (70.3 %)
Q21: Had trouble sleeping	5 (2.1 %)	10 (4.2 %)	33 (14.0 %)	22 (9.3 %)	166 (70.3 %)
Q26: Had difficulty saying certain words	2 (0.8 %)	1 (0.4 %)	18 (7.6 %)	29 (12.3 %)	186 (78.8 %)
Q30: People had difficulty understanding what you were saying	4 (1.7 %)	7 (3.0 %)	25 (10.6 %)	20 (8.5 %)	180 (76.3 %)
Q32: Had difficulty keeping your teeth clean	17 (7.2 %)	27 (11.4 %)	78 (33.1 %)	36 (15.3 %)	78 (33.1 %)
Domain 3: Socio-emotional Well-Being					
Q12: Been unhappy or sad	6 (2.5 %)	13 (5.5 %)	43 (18.2 %)	23 (9.7 %)	151 (64.0 %)
Q16: Felt worried or anxious	6 (2.5 %)	7 (3.0 %)	47 (19.9 %)	31 (13.1 %)	145 (61.4 %)
Q20: Avoided smiling or laughing with other children	10 (4.2 %)	93 (3.8 %)	39 (16.5 %)	31 (13.1 %)	147 (62.3 %)
Q27: Felt that you look different	7 (3.0 %)	4 (1.7 %)	25 (10.6 %)	29 (12.3 %)	171 (72.5 %)
Q33: Been worried about what other people think	5 (2.1 %)	12 (5.1 %)	35 (14.8 %)	28 (11.9 %)	156 (66.1 %)
Q18: Felt shy or withdrawn	4 (1.7 %)	11 (4.7 %)	42 (17.8 %)	28 (11.9 %)	151 (64.0 %)
Q25: Been teased, bullied or called names by other children	4 (1.7 %)	8 (3.4 %)	18 (7.6 %)	22 (9.3 %)	184 (78.0 %)
Q23: Got angry	8 (3.4 %)	14 (5.9 %)	28 (11.9 %)	25 (10.6 %)	161 (68.2 %)
Domain 4: School/Environment					
Q13: Missed school	0 (0 %)	3 (1.3 %)	24 (10.2 %)	22 (9.3 %)	187 (79.2 %)
Q19: Had difficulty paying attention in school	1 (0.4 %)	10 (4.2 %)	21 (8.9 %)	22 (9.3 %)	182 (77.1 %)
Q24: Did not want to speak/read out loud in class	5 (2.1 %)	5 (2.1 %)	20 (8.5 %)	14 (5.9 %)	192 (81.4 %)
Q36: Did not want to go to school	9 (3.8 %)	6 (2.5 %)	21 (8.9 %)	17 (7.2 %)	183 (77.5 %)
Domain 5: Self-image					
Q14: Been reassured or put in trust through	97 (41.1 %)	23 (9.7 %)	63 (26.7 %)	36 (15.3 %)	17 (7.2 %)
Q31: Felt that you were good looking	7 (3.0 %)	14 (5.9 %)	46 (19.5 %)	25 (10.6 %)	144 (61.0 %)
Q39: Felt having healthy teeth	35 (14.8 %)	40 (16.9 %)	51 (21.6 %)	87 (36.9 %)	23 (9.7 %)
Q43: Felt good about himself	25 (10.6 %)	26 (11.0 %)	22 (9.3 %)	65 (27.5 %)	98 (41.5 %)
Q40: When I am older, I believe that I’ll have good teeth	37 (15.7 %)	46 (19.5 %)	55 (23.3 %)	72 (30.5 %)	26 (11.0 %)
Q41: When I am older, I believe that I will be healthy	20 (8.5 %)	46 (19.5 %)	51 (21.6 %)	82 (34.7 %)	37 (15.7 %)

### Reliability (*n* = 236)

Cronbach’s alpha for the global COHIP score was 0.880 and varied from 0.872 to 0.888 when either item 23 (‘got angry because of your teeth, mouth or face’) or item 14 (‘been reassured or put in trust through your teeth, mouth or face’) was deleted. Cronbach’s alphas for each of the five subscales values were as follows: Oral Health = 0.700; Functional Well-Being = 0.664; Socio-emotional Well-Being = 0.846; School/Environment = 0.664; Self-image = 0.700.

### Concurrent validity (*n* = 236)

Results for concurrent validity are given Table 2. Lower COHIP scores were found for children with self-perceived poor (or very poor) general health (p-value < 0.0001), unsatisfactory oral health (p-value < 0.0001), dental problems (p-value <0.0001) and needs for dental treatment (p-value < 0.0001) (Table 2).

**Table 2:** Concurrent validity: COHIP scores and self-perception of oral and general health (mean values ± SD) (*n* = 236)

		Domain 1	Domain 2	Domain 3	Domain 4	Domain 5	Global Score
Self-perception of general health	Excellent (*n* = 32)	28.80 ± 4.87	21.39 ± 2.27	28.23 ± 4.96	14.78 ± 2.45	15.61 ± 4.28	108.81 ± 13.56
	Good (*n* = 84)	28.20 ± 5.89	20.32 ± 3.33	27.88 ± 5.40	14.99 ± 1.85	15.72 ± 3.98	107.12 ± 15.79
	Moderate (*n* = 79)	25.89 ± 6.04	19.47 ± 3.24	26.21 ± 5.59	14.14 ± 2.41	12.31 ± 4.37	98.02 ± 13.87
	Poor (*n* = 11)	26.09 ± 6.57	18.53 ± 4.22	26.43 ± 5.07	13.00 ± 2.61	13.51 ± 5.71	97.56 ± 18.10
	Very poor (*n* = 7)	24.82 ± 9.66	14.71 ± 6.85	20.67 ± 8.17	10.95 ± 4.88	8.09 ± 2.06	79.24 ± 26.79
	*Sig.*	*0.045*	*<0.0001*	*0.006*	*<0.0001*	*<0.0001*	*<0.0001*
Satisfied with oral health	Yes (*n* = 118)	28.68 ± 5.48	20.62 ± 3.30	28.97 ± 4.01	14.74 ± 2.23	15.96 ± 3.65	108.96 ± 13.84
	No (*n* = 81)	24.53 ± 6.79	18.63 ± 4.21	23.90 ± 6.94	13.78 ± 2.75	11.55 ± 4.63	92.40 ± 17.81
	*Sig.*	*<0.0001*	*0.001*	*<0.0001*	*0.018*	*<0.0001*	*<0.0001*
Presence of dental problems (Frequency)	Never (*n* = 12)	29.21 ± 5.50	20.63 ± 3.34	28.65 ± 4.15	14.80 ± 2.07	15.00 ± 4.47	108.28 ± 13.72
	Seldom (*n* = 104)	25.66 ± 6.09	19.42 ± 3.63	25.36 ± 6.48	14.08 ± 2.65	12.65 ± 4.78	97.17 ± 16.65
	Sometimes (*n* = 4)	17.69 ± 4.57	15.50 ± 3.70	22.75 ± 7.09	12.67 ± 4.11	11.75 ± 2.75	80.35 ± 14.32
	Always (*n* = 2)	13.50 ± 3.54	9.00 ± 5.66	11.50 ± 3.54	7.50 ± 4.95	8.00 ± 2.83	49.50 ± 20.51
	*Sig.*	*<0.0001*	*<0.0001*	*<0.0001*	*<0.0001*	*<0.0001*	*<0.0001*
Need for dental care	No (*n* = 97)	29.06 ± 5.76	21.05 ± 2.64	29.10 ± 3.43	14.76 ± 1.91	15.31 ± 4.29	109.28 ± 12.82
	Yes (*n* = 121)	25.51 ± 5.71	18.97 ± 4.07	25.07 ± 6.48	14.17 ± 2.73	12.79 ± 4.60	96.51 ± 16.53
	*Sig.*	*<0.0001*	*<0.0001*	*<0.0001*	*0.021*	*<0.0001*	*<0.0001*

### Known groups and discriminant validity (*n* = 236)

The relationships between COHIP scores and various socio-demographic and clinical variables are presented Table 3. The COHIP scores were statistically significantly related to the ethnic group, school attendance and gender (p-value < 0.05). Moreover, participants with tartar or infectious processes experienced higher OHRQoL impacts when compared with those without (p-value < 0.05). In the same way, participants with at least one decayed, missing or filled permanent tooth (D3MFT > 1) experienced lower COHIP scores than those with no dental decay (p-value < 0.05). It should be noted that in the sample, untreated carious lesions represented more than 70 % of the DMFT score. The COHIP scores did not vary significantly depending on the presence of gingivitis, mucosal lesions, untreated fractured permanent anterior teeth or dental plaque. Participants with a clinically attested urgent need for dental treatment (level 3 COCNI) presented higher impacts on their OHRQoL (p-value < 0.05). High dental fear (DF VAS >6) was related to lower COHIP scores (p-value < 0.05). Participants who had already visited a dentist scored better OHRQoL (p-value < 0.05). Discriminant validity is explored Table 4 with the calculation of correlation coefficients between COHIP scores and clinical variables. Low but often significant correlation coefficients were observed with higher correlations being found for the oral health dimension.

**Table 3:** Relationship between COHIP scores, dental and socio-demographic variables (mean values ± SD) (*n* = 236)

		Domain 1	Domain 2	Domain 3	Domain 4	Domain 5	Global Score
Ethnic group	Melanesian (*n* = 54)	27.17 ± 6.24	19.34 ± 3.78	26.96 ± 5.45	14.10 ± 2.57	14.03 ± 4.85	101.61 ± 16.56
	Caledonian (*n* = 117)	26.66 ± 6.15	19.61 ± 4.05	27.07 ± 5.50	14.20 ± 2.64	13.64 ± 4.70	101.17 ± 16.56
	Polynesian (*n* = 39)	27.27 ± 6.53	20.37 ± 2.55	25.44 ± 6.90	14.61 ± 2.35	12.65 ± 4.80	100.34 ± 18.64
	other (*n* = 18)	28.46 ± 6.21	21.83 ± 1.92	28.74 ± 4.06	15.89 ± .47	16.14 ± 3.88	111.06 ± 12.34
	*Sig.*	*0.698*	*0.001*	*0.173*	*<0.0001*	*0.040*	*0.026*
School attendance	Internal (*n* = 8)	28.16 ± 5.12	20.70 ± 2.69	27.62 ± 3.67	14.46 ± 1.84	12.95 ± 3.57	103.90 ± 11.80
	Half boarder (*n* = 196)	27.18 ± 6.04	20.20 ± 3.36	27.18 ± 5.66	14.68 ± 2.18	13.92 ± 4.83	103.16 ± 15.87
	External (*n* = 31)	25.85 ± 7.46	17.50 ± 4.86	24.84 ± 6.65	12.48 ± 3.46	12.78 ± 4.53	93.45 ± 21.56
	*Sig.*	*0.473*	*<0.0001*	*0.105*	*<0.0001*	*0.416*	*0.011*
Sex	Boys (*n* = 106)	27.56 ± 5.92	20.08 ± 3.41	28.12 ± 4.70	14.36 ± 2.61	14.60 ± 4.67	104.73 ± 15.89
	Girls (*n* = 130)	26.63 ± 6.40	19.70 ± 3.88	25.87 ± 6.35	14.41 ± 2.37	13.05 ± 4.72	99.66 ± 17.30
	*Sig.*	*0.251*	*0.436*	*0.003*	*0.901*	*0.012*	*0.021*
Calculus^d^	Absence (*n* = 168)	27.62 ± 6.11	20.19 ± 3.35	27.14 ± 5.66	14.52 ± 2.29	14.23 ± 4.73	103.70 ± 16.39
	Presence (*n* = 67)	25.63 ± 6.26	19.07 ± 4.31	26.16 ± 6.03	14.02 ± 2.88	12.56 ± 4.66	97.44 ± 17.36
	*Sig.*	*0.026*	*0.035*	*0.244*	*0.160*	*0.015*	*0.010*
D3MFT^a^	0 (*n* = 128)	27.44 ± 6.12	20.53 ± 3.17	27.31 ± 5.63	14.77 ± 1.99	13.90 ± 4.91	103.96 ± 16.34
	≥1 (*n* = 108)	26.58 ± 6.29	19.09 ± 4.07	26.38 ± 5.92	13.93 ± 2.89	13.56 ± 4.57	99.54 ± 17.18
	*Sig*	*0.291*	*0.002*	*0.215*	*0.009*	*0.582*	*0.044*
Infectious dental process^b^	No (*n* = 205)	27.56 ± 6.14	19.96 ± 3.62	27.22 ± 5.55	14.51 ± 2.33	13.89 ± 4.80	103.13 ± 16.29
	Yes (*n* = 31)	23.65 ± 5.51	19.31 ± 4.01	24.68 ± 6.77	13.56 ± 3.20	12.83 ± 4.35	94.03 ± 18.47
	*Sig.*	*0.001*	*0.361*	*0.022*	*0.046*	*0.248*	*0.005*
COCNI index^c^	Risk factors (*n* = 37)	29.03 ± 5.84	21.21 ± 2.28	27.83 ± 5.27	14.97 ± 1.57	15.05 ± 4.55	108.09 ± 14.47
	Dental needs (*n* = 100)	27.66 ± 6.13	19.87 ± 3.69	27.22 ± 5.33	14.55 ± 2.45	13.74 ± 4.88	103.04 ± 16.32
	Urgent needs (*n* = 96)	25.56 ± 6.09	19.40 ± 3.97	26.09 ± 6.39	13.99 ± 2.76	13.32 ± 4.60	98.36 ± 17.50
	*Sig*	*0.006*	*0.039*	*0.224*	*0.041*	*0.153*	*0.006*

^a^Number of decayed, missing of filled permanent teeth, carious threshold = stage 3 or 4 of Ekstrand’s classification [30]

^b^Presence of an abscess, a tooth with pulpal exposure or an apical fistula

^c^Clinical Oral Care Needs Index [34]

^d^Presence of calculus on a group of teeth, all the sextants of one arch or all sextants of two arches [32]

**Table 4:** Discriminant Validity: Correlation between COHIP scores and clinical variables (*n* = 236)

Partial Spearman correlation adjusted for gender	Calculus^c^	Infectious dental process^b^	D3MFT^a^	COCNI index^d^
Domain 1	-.146^*^	-.213^**^	-.057	-.212^**^
Domain 2	-.137^*^	-.060	-.165^*^	-.134^*^
Domain 3	-.076	-.149^*^	-.063	-.124
Domain 4	-.092	-.130^*^	-.135^*^	-.135^*^
Domain 5	-.158^*^	-.075	-.080	-.088
COHIP Score	-.168^**^	-.183^**^	-.186^**^	-.195^**^

*p < 0.05; **p < 0.01

^a^Number of decayed, missing of filled permanent teeth, carious threshold = stage 3 or 4 of Ekstrand’s classification [30]

^b^Presence of an abscess, a tooth with pulpal exposure or an apical fistula

^c^Presence of calculus on a group of teeth, all the sextants of one arch or all sextants of two arches [32]

^d^Clinical Oral Care Needs Index [34]

### Factor analysis (*n* = 236)

Results for factor analysis are given Table 5. Principal component analysis revealed the presence of eight components with eigenvalues exceeding 1, explaining 23.5, 7.2, 5.6, 5.1, 3.9, 3.8, 3.7 and 3.2 % of the variance respectively. An inspection of the screeplot revealed a clear break after the fourth component. Using Cattell’s scree test [37], it was decided to retain four components for further investigation.

**Table 5:** Factor Analysis: Rotated Component matrix (*n* = 236)

	Component			
	1	2	3	4
Domain 1: Oral health				
Q1: Had pain in your teeth/toothache			0.366	
Q2: Been breathing through your mouth or snoring			0.345	
Q3: Had discoloured teeth or spots on your teeth			0.524	
Q4: Had crooked teeth or spaces between your teeth			0.470	
Q5: Had sores/sore spots in or around your mouth			0.548	
Q6: Had bad breath			0.692	
Q7: Had bleeding gums			0.432	
Q8: Had food sticking in or between your teeth			0.558	
Q9: Had pain or sensitivity in teeth with hot/cold things			0.395	
Q10: Had dry mouth or lips			0.301	
Domain 2: Functional Well-Being				
Q11: Had trouble biting/chewing apple, carrot/firm meat		0.618		
Q15: Had difficulty eating foods you would like to eat		0.669		
Q21: Had trouble sleeping		0.638		
Q26: Had difficulty saying certain words		0.640		
Q30: People had difficulty understanding what you were saying		0.564		
Q32: Had difficulty keeping your teeth clean			0.434	
Domain 3: Socio-emotional Well-Being				
Q12: Been unhappy or sad	0.546			
Q16: Felt worried or anxious	0.579			
Q20: Avoided smiling or laughing with other children	0.638			
Q27: Felt that you look different	0.639			
Q33: Been worried about what other people think	0.696			
Q18: Felt shy or withdrawn	0.661			
Q25: Been teased, bullied or called names by other children	0.625			
Q23: Got angry	0.609			
Domain 4: School/Environment				
Q13: Missed school		0.545		
Q19: Had difficulty paying attention in school	0.480			
Q24: Did not want to speak/read out loud in class	0.537			
Q36: Did not want to go to school		0.602		
Domain 5: Self-image				
Q14: Been reassured or put in trust through				0.445
Q31: Felt that you were good looking	0.602			
Q39: Felt having healthy teeth				0.680
Q43: Felt good about himself				0.603
Q40: When I am older, I believe that I’ll have good teeth				0.713
Q41: When I am older, I believe that I will be healthy				0.683

Extraction Method: Principal Component Analysis

Rotation Method: Varimax with Kaiser Normalisation

This was further supported by the results of parallel analysis, which showed only four components with eigenvalues exceeding the corresponding criterion values for a randomly generated data matrix of the same size (34 variables and 236 respondents).

To aid in the interpretation of these four components, Varimax rotation was performed. The rotated solution revealed the presence of a simple structure, with components showing a number of strong loadings and all variables loading substantially on only one component. The four components solution explained a total of 41.5 % of the variance, with component 1 contributing 13.5 %, component 2 contributing 11.4 %, component 3 contributing 9.4 %, component 4 contributing 7.2 %. Social-emotional well-being and some school environment items loaded on component 1, functional well-being items loaded strongly on component 2 with some school environment items, oral health items loaded on component 3, and self-image items loaded strongly on component 4 (Table 5).

### Reproducibility (*n* = 60)

The test–retest reliability of the overall COHIP was excellent (ICC = 0.904; p-value < 0.0001) and for the domains of oral health (ICC = 0.829; p-value < 0.0001), functional well-being (ICC = 0.882; p-value < 0.0001), social-emotional well-being (ICC = 0.900; p-value < 0.0001), school environment (ICC = 0.760; p-value < 0.0001) and self-image (ICC = 0.842; p-value < 0.0001).

## Discussion

The results indicate that the impact of oral diseases on the OHRQOL of 12-year-old children in New Caledonia was high; 12 % claimed to have missed school and 40 % to have suffered from pain due to dental problems in the last 3 months. The French 34-items version of the COHIP exhibited acceptable validity and reliability, supporting its use for child populations of similar age in France. However, considering the ethnic distribution of the sample and the specificities of the dimensional structure of the COHIP, further studies should be designed to verify the impact of ethnicity on the validity of the COHIP and to explore more thoroughly the structure of the French version.

The strength of this study is that the validation process was conducted in a sample that can be considered as representative of the population of Caledonian 12-year-old children. The risk of selection bias is limited, as convenience samples were used only at start for the clarification of the wording and for the test-retest procedure. For the main validation procedure, children were randomly selected (one child in four in each cluster) from a national representative sample of New Caledonian schoolchildren. The participation rate was 80 %, which is usually considered to be satisfactory, given that the COHIP questionnaires were self-administered. Nevertheless, the risk of selection bias cannot be totally excluded; families of children with poor oral health and high dental needs may have been less likely to return the written consents.

The COHIP is one of the most frequently used of the OHRQOL indexes in children; it was found to be a high quality instrument [38]. A comparison of the psychometric properties of the COHIP and OHIP-14 has been conducted among Iranian adolescents; The COHIP was considered as preferable as it was able to identify more impacts [39]. In this study, the COHIP was effectively able to explore the various impacts of oral diseases with, for example, 26 % of the children claiming to have difficulty in chewing food due to dental problems. Another study has assessed the methodological quality of the development and testing of the CPQ, Child-OIDP and COHIP questionnaires. It appeared that the three tools have been used successfully in epidemiological studies and that the COHIP employed the most rigorous development strategy [38]. Slade and Reisine concluded that the COHIP needed to be tested in more community samples in order to establish its ability to evaluate clinically meaningful differences between oral health conditions [27]. In this field, the association between the COCNI Index and the COHIP scores indicated that children with urgent needs had COHIP scores lower than 100.

The Cronbach alpha coefficient indicated excellent internal consistency for the overall COHIP score and demonstrated the homogeneity of items, as has already been verified in previous English, Dutch, Korean and Persian versions [18, 22, 23, 40]. Consistency was moderate for functional Well-Being (0.66) and satisfactory for the Socio-emotional Well-Being (0.85).

The test-retest findings suggested very good reproducibility for the overall COHIP (ICC = 0.904) and for each of the underlying subscales (ICC >0.760). Values greater than or equal to 0.7 are considered acceptable. This finding was similar to that reported for the English version [18].

Evaluation of the concurrent validity was based on the support of theoretical relationships between the COHIP and other questionnaires that assess similar constructs. Concurrent validity was performed with the expected associations between the COHIP scores and the reported oral and general health status, perceived need for dental treatment and self-satisfaction with oral health. Lower COHIP scores were associated with poorer self-perceived oral and general health, greater need for dental care and low satisfaction with oral health. This testing was consistent with other reports in the literature [19, 39].

The findings also demonstrate that the COHIP was able to discriminate between children with different clinical conditions. Individuals with a satisfactory dental status had better quality of life scores compared with those with more severe conditions. Discriminant validity testing on known groups revealed lower OHRQoL scores for children with deprived social status, unhealthy oral behaviours or high levels of dental fear [18]. Moreover, this study revealed that children with treatment needs (as measured by the COCNI index) exhibited lower COHIP scores. In previous validation surveys, the discriminant analysis was conducted by comparing COHIP scores between orthodontic, paediatric or cleft palate child populations [40, 41] or by comparing scores between children with or without dental caries or orthodontic needs [21, 25]. Because the COHIP was previously developed and validated using a cleft palate population, it was more sensitive in measuring treatment needs of adolescents with cleft palate; the present study has explored other aspects of oral status, through evaluation using the COCNI index.

In our study, the principal-components factor analysis identified a four-dimensional structure of the questionnaire with the school environment items being spread in two different components related to socio-emotional and functional well-being. Two questions from the oral health and self-image dimensions also were found to be related to a different component. The structure observed in the NC study was thus not completely consistent with the initial COHIP dimensional structure [34]. During the initial processes of validation in Canada and France, the number of French children involved was low, which did not allow a complete evaluation of the French version. Moreover, the cultural context of NC may also explain the slight differences observed in the factor analysis. This shows that it could be interesting to re-explore the final choice of the items to be kept in the French version and to evaluate the impact of cultural characteristics on the COHIP structure.

The present study revealed that dental diseases impacted greatly the quality of life of Caledonian children. The COHIP mean score (101.9) was slightly higher than observed in USA and Canada (99) and lower than in Korea, Iran or Netherlands [18, 22, 23, 41]. The situation of Caledonian children was more favourable than that of children with craniofacial conditions in the Netherlands or North America [21, 24, 42].

In the present survey, participants from different ethnic groups exhibited various oral impacts. In New Caledonia, social status and educational level are strongly associated with ethnicity; Kanak (the original inhabitants of New Caledonia) and Polynesian people (Wallisians essentially) have lower educational and income levels than white Europeans (Caledonians and Metropolitan French) [43]. These results cannot be compared with previous findings showing that the social situation influences the level of the impacts of dental diseases on oral health [44]. Indeed, the influence of ethnic cultural differences cannot easily be separated from the social status of the different groups, which were compared.

## Conclusion

This study demonstrates that the French 34-items version of the COHIP is a valid measure and is appropriate for measuring children’s OHRQoL in France. It has satisfactory psychometric properties but further research is required to evaluate its sensitivity, specificity and its ability to detect clinically important changes over time in children. This study also provided useful data about the OHRQoL of Caledonian children that will help public health providers to develop an Oral Health Promotion program in the NC territory.

## Abbreviations

NC: New Caledonia
OHRQoL: Oral health related quality of life
COHIP: Child Oral Health Impact Profile
CPQ: Child perception questionnaire
ECOHIS: Early Childhood Oral Health Impact Scale
COCNI: Clinical Oral Care Needs Coefficients Index
ICC: Intraclass correlation
OIDP: Oral Impacts on Daily Performances
D3: Dentine threshold for carious lesions
D_3_MFT: Number of decayed, missing or filled permanent teeth
D_3_T-Mo: Number of decayed first permanent molars
